# A Lytic *Mosigvirus* Phage (GADS24) from a Poultry-Farm Environment: Genome-Resolved Characterization and In Vitro Biocontrol-Relevant Phenotyping Against *Escherichia coli*

**DOI:** 10.3390/ijms27031276

**Published:** 2026-01-27

**Authors:** Jehan Alrahimi, Ghadah Alsubhi, Alia Aldahlawi, Fatemah S. Basingab, Mohammed A. Imam, Hashim Felemban, Najwa Alharbi, Sana Alshaik, Hala S. Sonbol, Kawther Zaher, Esam I. Azhar

**Affiliations:** 1 Department of Biological Sciences, Faculty of Sciences, King Abdulaziz University, Jeddah 22254, Saudi Arabia; 2 Immunology Unit, King Fahd Medical Research Center, King Abdulaziz University, Jeddah 22254, Saudi Arabia; 3 Department of Medical Microbiology and Parasitology, Faculty of Medicine at Al-Qunfudah, Umm Al-Qura University, Al-Qunfudah 21961, Saudi Arabia; 4 Department of Medical Laboratory Sciences, Faculty of Applied Medical Sciences, King Abdulaziz University, Jeddah 22254, Saudi Arabia; 5 Special Infectious Agents Unit, King Fahd Medical Research Center, King Abdulaziz University, Jeddah 22254, Saudi Arabia; 6 Microbiology Department, Science College, King Abdulaziz University, Jeddah 22254, Saudi Arabia; 7 Biochemistry Department, Faculty of Science, King Abdulaziz University, Jeddah 22254, Saudi Arabia

**Keywords:** bacteriophage, *Escherichia coli*, *Mosigvirus*, *Tevenvirinae*, lytic phage, genome sequencing, comparative genomics, host range, poultry farm, biocontrol

## Abstract

Multidrug-resistant (MDR) *Escherichia coli* (*E. coli*) at the poultry–human interface motivates evaluating strictly lytic bacteriophages as targeted biocontrol candidates. A lytic *E. coli* phage (GADS24) was isolated from poultry waste in Saudi Arabia. Plaque formation and host range were assessed against 10 clinical *E. coli* isolates. Virion morphology was examined by transmission electron microscopy (TEM). Whole-genome sequencing (Illumina) and annotation (Prokka/RAST) were followed by comparative genomics (BLASTn 2.15.0, ANI JSpeciesWS: 2014–2025 Ribocon GmbH—Version: 5.0.3, dDDH GGDC: GGDC 3.0 and phylogenetic/proteomic analyses for taxonomic placement. GADS24 formed clear plaques and lysed 5 of 10 clinical *E. coli* isolates tested. TEM revealed an icosahedral capsid (~72.6 nm) and a contractile tail (~131.7 nm), consistent with *Tevenvirinae*/*Mosigvirus* morphology. The dsDNA genome is 168,896 bp (GC 43.8%) with 268 predicted ORFs and two tRNA genes (tRNA-Arg and tRNA-Met); no lysogeny-related genes were detected. The closest relative was *Escherichia* phage JN02 (98.44% ANI; 57.8% dDDH), supporting assignment to *Mosigvirus* while indicating a genome-resolved distinct lineage. The genome is available in GenBank (OQ703618). GADS24 represents a genome-resolved, strictly lytic *Mosigvirus* with in vitro biocontrol-relevant phenotyping against *E. coli*, supporting follow-up development for poultry-associated infection control and deeper phage–host interaction studies.

## 1. Introduction

*Escherichia coli* (*E. coli*) is a Gram-negative, facultative anaerobe that commonly inhabits the gastrointestinal tract of humans and animals. While many strains are harmless commensals, pathogenic variants can cause intestinal disease and a wide range of extraintestinal infections, including urinary tract infections, neonatal meningitis, bacteremia, pneumonia, and gastroenteritis [1,2]. The public health impact of *E. coli* is amplified by its genomic plasticity and propensity to acquire virulence determinants and antimicrobial resistance (AMR) genes via horizontal gene transfer, enabling rapid adaptation across community, healthcare, and agricultural settings [1,2]. Consistent with this, antibiotic-resistant *E. coli* contributes substantially to the AMR burden and is recognized by public health agencies as a significant and persistent threat that increases morbidity, mortality, and healthcare costs [3,4]. Poultry production systems, including farm environments and associated waste streams, can act as reservoirs and dissemination hubs for MDR *E. coli*, facilitating persistence and potential transfer along the poultry–human interface. Accordingly, phages isolated from poultry-associated niches may represent ecologically relevant candidates for targeted biocontrol approaches in food-production settings.

These pressures have renewed interest in bacteriophages (phages) as precision antimicrobials. Phages are abundant across natural environments and can be isolated from ecological niches where target bacteria circulate, including animal production systems and their waste streams [5]. In contrast to broad-spectrum antibiotics, phages can provide targeted killing with reduced disruption of the commensal microbiota, and their capacity to replicate at sites of infection can amplify their biologic effect when susceptible hosts are present [4,6,7]. At the same time, phage deployment is not without challenges: therapeutic success depends on phage–host compatibility (often shaped by bacterial receptors and phage adsorption machinery), and bacterial escape can occur through resistance or ecological context effects. This has motivated current strategies that emphasize rational phage selection, host range profiling, and—in many settings—cocktail-based approaches to broaden coverage while limiting the emergence of resistance [8,9,10]. Evidence syntheses of clinical and safety trials also highlight that rigorous characterization and transparent reporting are essential for translating phage candidates into reproducible interventions [11].

Within One Health-relevant contexts, poultry production is an important interface for *E. coli* circulation, including strains with resistance traits and zoonotic potential. Phage biocontrol has therefore been explored as a complementary measure to reduce bacterial loads in foods, processing environments, and animal-associated reservoirs [12,13]. Several studies have reported lytic *E. coli* phages active against antibiotic-resistant or pathogenic strains, supporting the feasibility of isolating candidates for biocontrol-oriented development [14,15,16,17]. However, despite the accelerating pace of phage discovery, a significant fraction of environmental phage diversity remains genomically unresolved, and many lineages contain extensive “hypothetical” coding capacity with unclear implications for host range, fitness, and application readiness. This knowledge gap is particularly relevant for large dsDNA phages within the tailed-phage radiation, where genome-resolved analyses increasingly underpin modern taxonomy and facilitate safer downstream use through screening for undesirable genetic cargo [18].

Here, we report the isolation of a strictly lytic *E. coli* phage, GADS24, recovered from a poultry-farm environment and characterized with an application-oriented focus: plaque phenotype and high-titer propagation, host range across a panel of clinical *E. coli* isolates, virion morphology by transmission electron microscopy, and genome-resolved classification supported by comparative genomics and phylogenomic analyses. Using the current ICTV framework for bacterial viruses, we place GADS24 within the *Mosigvirus* genus (*Straboviridae*; *Tevenvirinae*), and provide a genomic resource to support subsequent work on phage–host interactions and on the implementation of biocontrol against *E. coli* across agricultural-to-clinical transmission landscapes.

## 2. Results

### 2.1. Isolation, Plaque Morphology, and Propagation of GADS24

**Figure 1:**
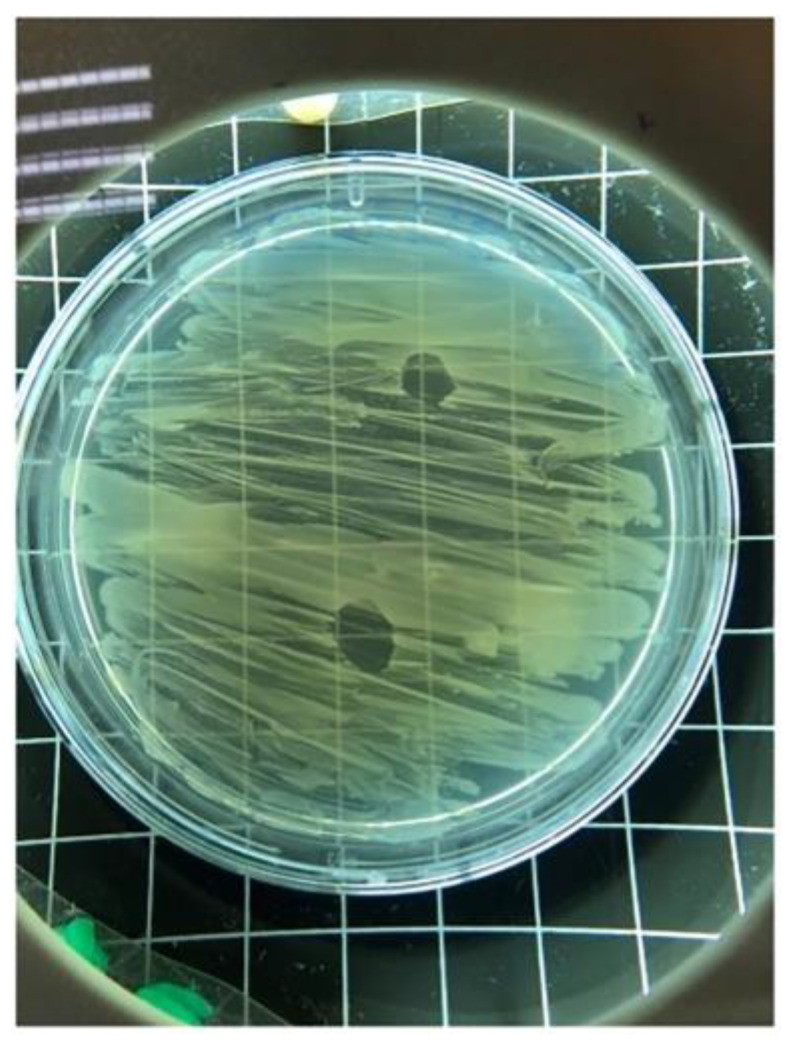
Spot assay showing clear, circular lysis zones produced by phage GADS24 on *E. coli* lawns.

**Figure 2:**
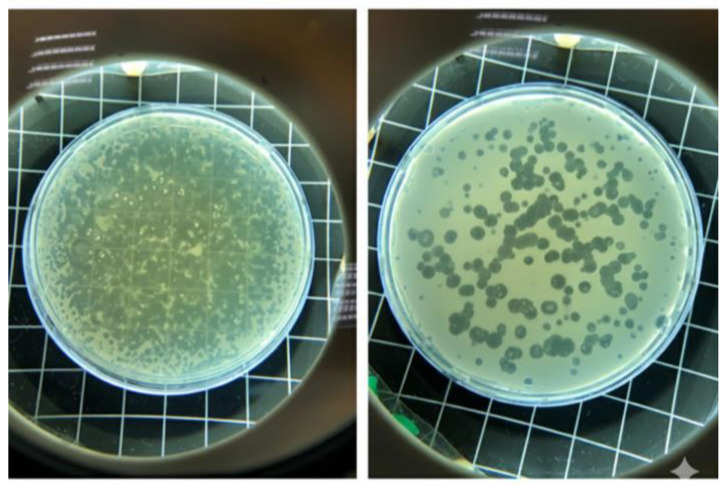
Plaque assay showing serial dilutions of phage GADS24. Small, clear plaques (<1 mm) were visible at high dilutions.

A lytic *E. coli* phage (GADS24) was recovered from poultry-farm environmental material and enriched on *E. coli* host cells (*E. coli* NRT114). Spot testing and double-layer plaque assays produced well-defined, clear lysis zones/plaques, with clear circular plaques of approximately ~1 mm under the tested conditions (Figure 1). High-titer lysates were obtained following propagation at 37 °C, reaching 4 × 10^8^ PFU/mL (Figure 2), and were used for downstream phenotyping and genome-resolved analyses.

### 2.2. Host Range Against Clinical E. coli Isolates

Host-range screening was performed against a panel of 10 non-duplicate clinical *E. coli* isolates provided by collaborators at King Abdulaziz University/King Fahd Medical Research Center (Jeddah, Saudi Arabia). Isolates were classified as multidrug-resistant (MDR) by the supplying laboratory using established clinical breakpoints and an accepted MDR definition (i.e., non-susceptibility to at least one agent in ≥3 antimicrobial categories). Lytic activity was recorded qualitatively as presence/absence of lysis (+/−) under the tested conditions (Table 1), indicating a moderate spectrum within the evaluated panel and supporting potential utility in targeted biocontrol or in rational phage-cocktail design. Quantitative efficiency-of-plating (EOP) was not determined in this initial report and will be included in follow-up optimization studies.

### 2.3. Virion Morphology by Transmission Electron Microscopy

**Figure 3:**
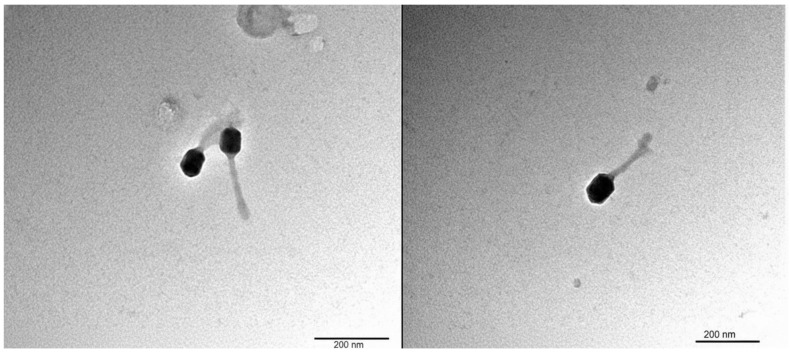
Transmission electron micrograph (TEM) of bacteriophage GADS24 showing an icosahedral head and a contractile tail (myovirus-like morphology). The phage particles were negatively stained with 3% uranyl acetate and imaged using a JEOL 1400 transmission electron microscope. Scale bar: 500 nm.

Transmission electron microscopy revealed an icosahedral capsid and a long contractile tail. The head diameter was approximately 72.6 nm, and the tail length was 131.7 nm, consistent with *Tevenvirinae*-like myoviruses and supporting placement within *Mosigvirus* (*Straboviridae*) (Figure 3) [18].

### 2.4. Genome Features and Phylogenomic Placement

**Figure 4:**
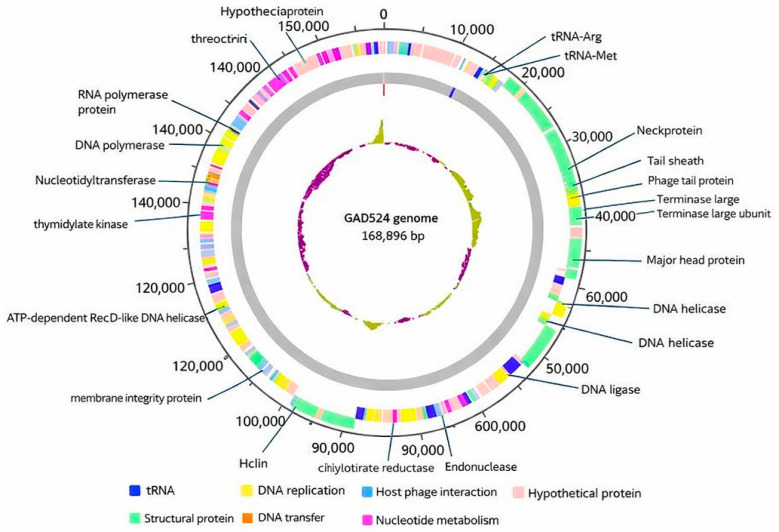
Circular genome map of bacteriophage GADS24 (168,896 bp; 268 predicted ORFs). Functional categories are color-coded: virion structure (green), host–phage interaction (light blue), nucleotide metabolism (fuchsia), DNA replication/repair (yellow), RNA-related functions (navy), and hypothetical proteins (pink).

Whole-genome sequencing produced a complete dsDNA genome (168,896 bp; GC 43.8%) with 268 predicted open reading frames and two tRNA genes (tRNA-Arg, GADS24_28; 12,211–12,287; −strand; and tRNA-Met, GADS24_29; 12,291–12,365; −strand) (Figure 4; Supplementary Table S1). Supplementary Table S1 (uploaded as a standalone file) contains the complete annotation of all predicted ORFs, including coordinates, strand orientation, and functional assignments.

**Figure 5:**
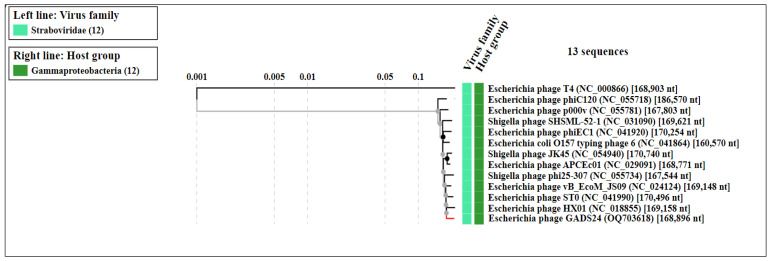
Whole-genome BLASTn-based phylogenetic tree showing the relationship of GADS24 to the top BLASTn-matched *Escherichia* phages (including ST0, HX01, and JN02).

**Figure 6:**
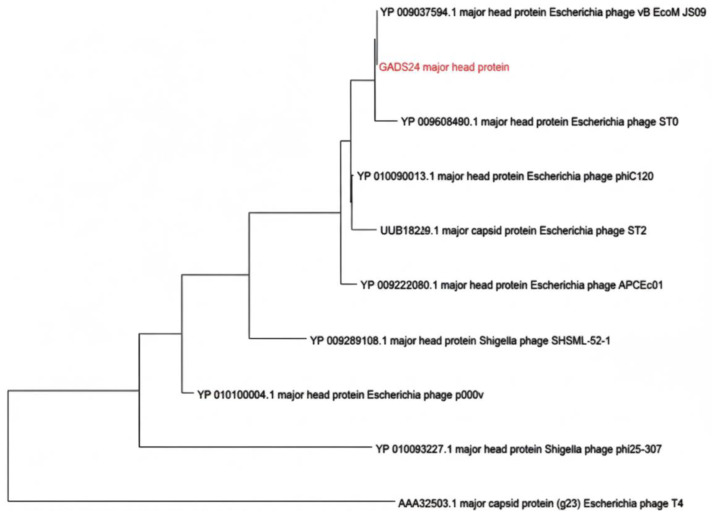
Whole-proteome (genome-wide) similarity tree generated using ViPTree (v3.3), showing the placement of GADS24 among related *Tevenvirinae* phages. GADS24 is highlighted and clusters nearest *Escherichia* phages ST0 and HX01.

No lysogeny-related genes were detected, consistent with a strictly lytic lifestyle. Comparative analyses using BLASTn, average nucleotide identity (ANI), and digital DNA–DNA hybridization (dDDH) identified close relatedness to *Mosigvirus* phages, including *Escherichia* phages ST0, HX01, and JN02. The closest match was phage JN02 (ANI 98.44%), whereas dDDH was 57.8%, supporting placement within *Mosigvirus* while suggesting a distinct species-level lineage under commonly used demarcation criteria (Figure 5 and Figure 6) [18].

**Genome-based safety screening:** To support biocontrol/therapeutic safety assessment, the GADS24 genome and predicted proteins were screened against dedicated antimicrobial resistance and virulence databases. No acquired antibiotic resistance determinants were detected in CARD/ResFinder-based searches, and no known virulence-factor genes (e.g., toxin-encoding cargo) were identified in virulence database screening. Consistent with a lytic lifestyle, no integrase/recombinase modules associated with lysogeny were observed. The genome encodes two tRNA genes (see Supplementary Table S1; tRNA-Arg and tRNA-Met), which may support efficient translation during infection and adaptation to host codon usage.

### 2.5. Functional Annotation and Gene Content

A total of 268 putative coding sequences were predicted; 141 were assigned putative functions spanning DNA replication and metabolism, virion morphogenesis/packaging, host–phage interaction, and lysis, while the remainder were annotated as hypothetical proteins (Figure 4; Supplementary Table S1). Genome-wide proteomic comparison using ViPTree supported the placement of GADS24 within the *Tevenvirinae* lineage and the *Mosigvirus* genus, clustering closest to *Escherichia* phages ST0 and HX01 (Figure 6), consistent with current ICTV taxonomy [18].

**Figure 7:**
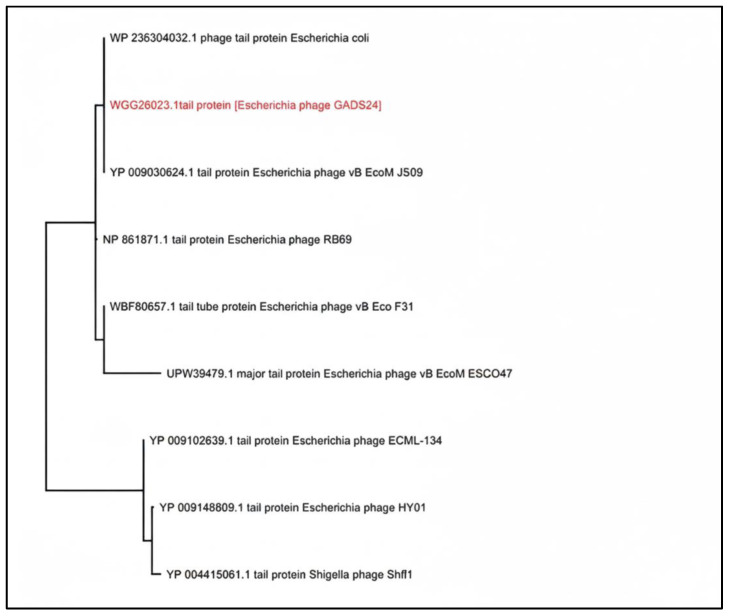
Maximum likelihood phylogenetic tree based on the amino acid sequence of the terminase large subunit of bacteriophage GADS24 and related *Tevenvirinae* phages. The tree was constructed in MEGA 11 using the JTT matrix-based model with 1000 bootstrap replicates. Bootstrap values (*n* = 1000) are indicated at branch nodes; GADS24 is highlighted.

To complement the genome-wide proteomic analysis (Figure 6), we constructed a maximum-likelihood phylogeny using the terminase large subunit, a widely used, conserved marker for tailed-phage classification. The resulting tree placed GADS24 closest to *Escherichia* phages HX01 and ST0, forming a distinct subbranch, supporting its designation as a separate species-level lineage within *Mosigvirus* (Figure 7). Consistent with this placement, GADS24 encodes a T4/RB69-like morphogenesis module typical of *Tevenvirinae* phages [19].

Genome-wide proteomic comparison using ViPTree confirmed that GADS24 clusters within the *Mosigvirus* genus and is most closely related to *Escherichia* phages ST0 and HX01 (Figure 6). This placement is consistent with the latest ICTV taxonomy for *Tevenvirinae*/*Straboviridae*, whose members typically possess large dsDNA genomes (~170–245 kb) encoding ~300–415 proteins [18].

**Figure 8:**
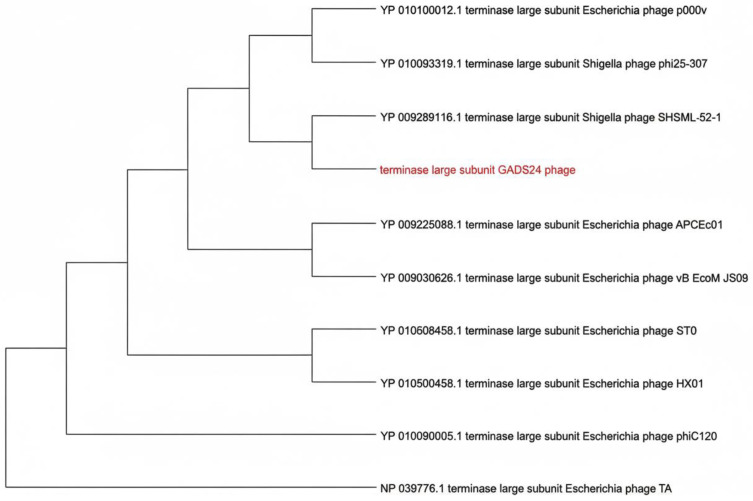
Whole-genome alignment of phage GADS24 with closely related phages ST0, HX01, and JN02, visualized using Easyfig.

To further examine genome organization and conservation, a whole-genome alignment was performed between GADS24 and its closest relatives (ST0, HX01, and JN02) using BLASTn, and the results were visualized with Easyfig. As shown in Figure 8, GADS24 exhibits high collinearity with related phages across the essential modules (DNA replication, virion morphogenesis, and lysis), whereas several regions diverge, including segments enriched for hypothetical proteins and putative host-interaction determinants, particularly in the latter. These differences support genomic divergence within the *Mosigvirus* lineage and highlight candidate regions for future functional investigation [20].

## 3. Discussion

Bacteriophages have broad applications in health, veterinary medicine, and industry [21]. The rapid emergence of MDR *Escherichia coli* poses a growing global threat and underscores the need for alternative antimicrobial strategies. In this context, phage therapy has re-emerged as a promising approach due to its high specificity, limited disruption of the host microbiota, and its ability to coevolve with bacterial targets [8,11,16,22,23,24]. Here, we report the isolation and genome-resolved characterization of GADS24, a strictly lytic *E. coli* phage recovered from a poultry farm environment in Saudi Arabia, and evaluate its in vitro activity against clinical *E. coli* isolates.

Transmission electron microscopy revealed a morphology typical of the *Tevenvirinae* lineage, with an icosahedral capsid and a long contractile tail, consistent with its placement within *Straboviridae* [25]. Host-range testing showed that GADS24 infected 5 of 10 clinical *E. coli strains*, indicating a moderate infectivity spectrum. While broader host-range phages may be advantageous in certain clinical or polymicrobial contexts, narrower host-range phages can offer greater specificity, reduce off-target effects, and help preserve commensal microbiota [10].

Genomic analysis showed that GADS24 possesses a double-stranded DNA genome of 168,896 bp with a GC content of 43.8%, encoding 268 predicted ORFs and two tRNA genes (tRNA-Arg and tRNA-Met) [18]. Of these ORFs, 141 were assigned predicted functions, including genes involved in DNA replication, virion structure and assembly, nucleotide metabolism, and host cell lysis, whereas 127 ORFs encoded hypothetical proteins, highlighting a substantial fraction of uncharacterized genetic content that warrants future functional investigation [13]. Comparative genomics and phylogenetic/proteomic analyses consistently place GADS24 within the *Mosigvirus* clade alongside related *Escherichia* phages (e.g., ST0, HX01, and JN02). While ANI values indicate close relatedness, the dDDH value below the commonly used species threshold supports the proposal that GADS24 represents a distinct genome-resolved lineage within *Mosigvirus*. Whole-genome alignment further indicates conserved synteny across major functional modules, with unique regions enriched for hypothetical proteins that may contribute to niche adaptation and host interaction [18]. Notably, a substantial fraction of predicted ORFs were annotated as hypothetical proteins. In genome-wide alignments, many of these hypothetical ORFs appear enriched within variable regions/genomic islands that interrupt otherwise conserved syntenic modules, consistent with the modular evolution typical of *Tevenvirinae* phages. Such islands often represent loci under strong selection, including host-range determinants, receptor-binding and tail-associated functions, and phage–host conflict systems (e.g., anti-restriction/anti-defense or transcriptional takeover proteins). Although definitive functions cannot be assigned without experimental validation, domain-based similarity searches (when detectable) and comparative placement across related *Mosigvirus* genomes suggest that at least some hypothetical proteins may contribute to host adaptation, niche specialization, or immune/defense evasion, whereas others may be lineage-specific to GADS24. Future work combining refined in silico domain profiling with transcriptomics/proteomics during infection will help prioritize candidates for functional characterization.

From a therapeutic/biocontrol perspective, GADS24 shows favorable features for downstream development: it is strictly lytic (no lysogeny-related modules were detected) and genome-based safety screening identified no acquired antibiotic resistance genes (CARD/ResFinder) and no known virulence-factor genes in dedicated database searches. In addition, the presence of phage-encoded tRNAs (*n* = 2) may reflect adaptation to translational demands and host codon usage, a feature reported in several large dsDNA-tailed phages, and does not by itself indicate biosafety risk.

This study is intended as an early-stage, genome-resolved characterization with in vitro phenotyping. Future work should prioritize functional interrogation of the hypothetical-protein repertoire (e.g., domain-based analyses and omics-guided validation), expanded host-range/EOP profiling, and application-oriented testing (e.g., killing curves and matrix-based assays), followed by appropriate in vivo efficacy and safety evaluation. Also, the absence of known virulence or antibiotic-resistance determinants supports a favorable biosafety profile for continued investigation. The moderate host range observed in the tested panel suggests potential utility for targeted biocontrol and provides a rationale for future cocktail design or expanded screening across broader *E. coli* diversity [24,25,26,27].

We acknowledge that adsorption kinetics and environmental stability (e.g., temperature, pH, and UV tolerance) are valuable for formulation and deployment. However, within the current revision timeframe, it was not feasible to conduct additional wet-lab assays such as classical one-step growth curves or extended environmental tolerance profiling. Accordingly, the present work is positioned as an early-stage, in vitro phenotyping and genome-resolved characterization study that provides a foundation for subsequent application-focused validation. Future studies should prioritize replication kinetics (adsorption, latent period, burst size), stability testing under relevant environmental conditions, and efficacy assessments in liquid time–kill assays and on appropriate matrices and surfaces (e.g., poultry-associated models), alongside broader host-range screening and in vivo validation to define pharmacodynamics, stability, and immune interactions. In addition, functional characterization of hypothetical proteins using transcriptomics/proteomics, formulation strategies for storage and delivery, and evaluation in phage cocktails or antibiotic–phage combinations may further enhance translational potential.

## 4. Materials and Methods

### 4.1. Bacterial Strain

The *E. coli* strain used in this study, identified as *E. coli* NRT114 (accession no. KP244263.1), was generously provided by Ms. Sanaa Alshaikh, a master’s student at King Abdulaziz University. The bacterial strain was cultured in Luria–Bertani (LB) broth composed of 5 g/L yeast extract, 10 g/L tryptone, and 5 g/L NaCl. The mixture was incubated at 29 °C with continuous shaking at 220 rpm. *Escherichia coli* strain NRT114 was used as the primary host for phage enrichment/isolation and routine propagation/titration, while the panel of 10 clinical *E. coli* isolates was used only for host-range screening (Section 4.5).

### 4.2. Phage Isolation

Environmental samples comprising soil, water, and chicken waste were collected from a poultry farm in Jeddah, Saudi Arabia. Ten milliliters of SM buffer (5.8 g NaCl, 2.0 g MgSO_4_·7H_2_O, 50 mL of 1 M Tris-HCl at pH 7.4, diluted to 1 L with distilled water) were added to the mixture. The suspension was centrifuged at 10,000× *g* for 10 min, and the supernatant was subsequently filtered through a 0.22 μm membrane filter. A 100 μL aliquot of the filtrate was added to a 5 mL log-phase *E. coli* culture, which was incubated overnight at 37 °C with shaking at 120 rpm. The culture was again centrifuged and filtered, and the resulting phage lysate was stored at 4 °C for further analysis [28]. To obtain a purified phage preparation for downstream analyses, the lysate was clarified/filtered, and phage particles were concentrated and purified by PEG 8000 precipitation followed by CsCl density-gradient purification, as described by Bonilla et al. [29].

### 4.3. Spot Assay

The lytic activity of the isolated phage was evaluated via the spot assay method described by Bonilla et al. [29]. 300 μL of mid-log-phase *E. coli* cells was mixed with 3 mL of semisolid LB agar and poured onto solidified LB agar plates to form a bacterial lawn. Subsequently, 10 μL of the phage suspension was spotted on the lawn in duplicate. The plates were incubated overnight at 37 °C, and plaque formation was recorded via a digital colony counter.

### 4.4. Plaque Assay and Phage Titration

Plaque assays were performed to determine the phage titer according to the protocol of Bonilla et al. [29]. An overnight culture of *E. coli* was diluted 1:100 in fresh LB broth and incubated at 37 °C with shaking at 200 rpm for approximately 2 h and 40 min. Serial tenfold dilutions of the phage lysate (10^−1^ to 10^−10^) were prepared using SM buffer. For each dilution, 700 μL was mixed with 300 μL of mid-log phase *E. coli* and 3 mL of molten semisolid agar, which was then overlaid onto LB agar plates. After overnight incubation at 37 °C, the plaque-forming units per milliliter (PFU/mL) were calculated via standard methods.

### 4.5. Host Range Determination

For host-range screening, ten non-duplicate clinical *E. coli* isolates were provided by collaborators at King Abdulaziz University/King Fahd Medical Research Center (Jeddah, Saudi Arabia). Isolates were confirmed as *E. coli* by standard biochemical identification and 16S rRNA gene sequencing. The supplying clinical laboratory classified these isolates as multidrug-resistant (MDR) using established clinical breakpoints and an accepted MDR definition (non-susceptibility to at least one agent in ≥3 antimicrobial categories). Host range was evaluated using a spot/overlay screening assay on bacterial lawns prepared by the double-layer agar method. Briefly, each isolate was grown to mid-log phase (OD600 ≈ 0.5–0.6), and 100 µL of culture was mixed with 3 mL of molten soft agar (0.6% agar, ~45 °C) and overlaid onto LB agar plates. After solidification, 10 µL of serially diluted phage suspension (10–10^−6^) was spotted onto the lawn and allowed to absorb. Plates were incubated overnight at 37 °C, and lysis was recorded qualitatively as complete clearing (+) or no clearing (−). For isolates showing lysis in spot tests, plaque formation was confirmed using the double-layer plaque assay under the same incubation conditions.

### 4.6. Transmission Electron Microscopy (TEM)

The morphology of the isolated phage was examined via transmission electron microscopy (TEM). A droplet of the PEG/CsCl-purified phage suspension (Section 4.2) was applied to Formvar/carbon-coated copper grids and allowed to adsorb for 2 min. The grids were then negatively stained with 3% uranyl acetate for 30 s, blotted, and air-dried. Observations were performed via a JEOL 1400 transmission electron microscope (JEOL Ltd., Tokyo, Japan) at the Core Facility of King Abdullah International Medical Research Center (KAIMRC), Riyadh.

### 4.7. Phage DNA Extraction

Genomic DNA was extracted from the PEG/CsCl-purified phage suspension prepared as described in Section 4.2 using the QIAamp^®^ DNA Mini Kit (Qiagen, Hilden, Germany) according to the manufacturer’s protocol. Briefly, 1000 µL of phage lysate was mixed with 180 µL of buffer ATL, vortexed, and incubated at 56 °C for 1 h. Then, 200 µL of buffer AL was added, followed by vortexing and a 10 min incubation at 70 °C. Subsequently, 200 µL of absolute ethanol was added, and then the mixture was transferred to a QIAamp spin column. The column was subsequently centrifuged at 8000 rpm for 1 min. Washing steps were performed using 500 µL of buffer AW1 and buffer AW2, with centrifugation at 8000 rpm and 14,000 rpm, respectively. Finally, the DNA was eluted with 60 µL of nuclease-free water and stored at −20 °C until further use.

### 4.8. Whole Genome Sequencing and Bioinformatics Analysis

Whole-genome sequencing was performed by MicrobesNG (Birmingham, UK; www.microbesNG.com (accessed 12 May 2024)). DNA libraries were prepared via the Nextera XT Library Prep Kit (Illumina, San Diego, CA, USA), according to the manufacturer’s instructions. Automated liquid handling was performed on the Hamilton Microlab STAR platform, and quantification of pooled libraries was performed using the KAPA Library Quantification Kit on a Roche LightCycler 96 system. Sequencing was performed on an Illumina HiSeq platform using a 250 bp paired-end read protocol.

The raw sequencing reads were quality-trimmed using Trimmomatic version 0.30 with a sliding-window quality threshold of Q15 [30]. De novo genome assembly was performed using SPAdes version 3.7 [31], and gene annotation was performed using Prokka [32].

For sequence-similarity searches, BLASTn/BLASTp analyses were performed using an E-value cutoff of 1 × 10^−5^. Unless otherwise stated, hits were considered significant when they met ≥70% query coverage and ≥30% amino-acid identity (BLASTp) or ≥70% nucleotide identity (BLASTn) for reporting close relationships. Functional annotations were assigned conservatively by integrating Prokka/RAST outputs with BLASTp domain similarity; only high-confidence matches meeting the above thresholds and consistent functional context (e.g., within conserved modules) were retained as “putative functions,” whereas ambiguous matches were reported as hypothetical proteins.

ARG/VFG and tRNA screening: To evaluate genome safety for biocontrol-relevant applications, predicted proteins and assembled contigs were screened for acquired antimicrobial resistance genes using CARD (RGI) and ResFinder (default identity/coverage thresholds; only hits meeting commonly accepted reporting cutoffs were considered). Putative virulence factor genes were assessed by sequence similarity searches against a curated virulence-factor database (e.g., VFDB), and hits were manually reviewed to exclude nonspecific matches to core phage structural/replication proteins. tRNA genes were identified from the assembled genome and cross-checked against the Prokka output; the complete list and coordinates are provided in Supplementary Table S1.

The annotated genome was visualized via Artemis release 18.2.0 [33]. Homology-based identification of coding sequences and functional annotation were performed via BLASTn and BLASTp against the NCBI nonredundant database [34]. Phylogenetic analysis of conserved structural and replication proteins, including the major capsid, tail fiber, and terminase large subunit, was conducted via ClustalW alignments in MEGA version 11 [35], employing the maximum likelihood method [36].

In addition, ViPTree version 3.3 was used to construct proteomic trees and to determine genome-wide relationships among closely related phages [37]. Multiple sequence alignments were generated using ClustalW with default parameters, and phylogenetic trees were inferred using maximum likelihood with 1000 bootstrap replicates; only bootstrap values ≥ 70% were interpreted as strongly supported.

## 5. Conclusions

This study reports the isolation and genome-resolved characterization of GADS24, a strictly lytic *Mosigvirus* phage recovered from a poultry farm environment and showing in vitro activity against clinical *E. coli* isolates. The combined phenotyping and genome-based classification support its potential utility in in vitro biocontrol-oriented applications and provide a foundation for future phage–host interaction studies.

Comparative genomics and phylogenetic analyses consistently placed GADS24 within the *Mosigvirus* clade, closely related to, but distinct from, reported members (including phages ST0 and HX01). Its moderate host range, together with the absence of lysogeny-associated genes and known virulence determinants, supports a favorable biosafety profile and highlights its potential relevance for therapeutic and agricultural exploration. Dedicated genome screening further supported biosafety considerations, with no acquired antimicrobial resistance genes (CARD/ResFinder) and no known virulence-factor genes detected in database-guided searches. Genome-wide synteny further indicated conservation of major functional modules with related phages, while unique genomic islands suggest evolutionary divergence and possible horizontal gene acquisition.

Given the escalating threat of antimicrobial resistance, GADS24 adds to the growing repertoire of lytic phages with clinical and agricultural relevance. It is observed that lytic activity against multiple *E. coli* isolates supports further investigation as a targeted biocontrol candidate to help reduce antibiotic reliance in food-production systems. A limitation of this study is that successive single-plaque purification (multiple rounds of plaque picking) was not performed; future work will include clonal purification alongside expanded phenotyping to strengthen confidence in sample homogeneity.

Future investigations should determine adsorption kinetics and replication parameters (latent period and burst size) using one-step growth experiments, evaluate environmental stability across relevant temperatures, pH ranges, and UV exposure, and pursue application-level validation through liquid killing-curve assays and testing on poultry-associated matrices/surfaces. Additional priorities include in vivo efficacy and safety evaluation, formulation and storage stability, and assessment of potential synergy with existing antimicrobials (e.g., antibiotics or phage cocktails).

## Figures and Tables

**Table 1:** Host-range screening of bacteriophage GADS24 against a panel of MDR clinical *E. coli* isolates (qualitative lysis outcome).

Clinical *E. coli* Strain *	Lytic Activity
*E. coli* Strain 53	+(Lysis observed)
*E. coli* Strain 33	+(Lysis observed)
*E. coli* Strain 16	+(Lysis observed)
*E. coli* Strain 30	−(No lysis)
*E. coli* Strain 21	+(Lysis observed)
*E. coli* Strain 9	−(No lysis)
*E. coli* Strain 401	−(No lysis)
*E. coli* Strain 882999	−(No lysis)
*E. coli* Strain 20	−(No lysis)
*E. coli* Strain 40	+(Lysis observed)

* The isolate panel represents clinical *E. coli* strains provided by collaborators at King Abdulaziz University/King Fahd Medical Research Center (Jeddah, Saudi Arabia). MDR classification was assigned by the supplying laboratory based on routine antimicrobial susceptibility testing and an accepted MDR definition (non-susceptibility to ≥1 agent in ≥3 antimicrobial categories). Isolate-level antibiograms and molecular pathotype assignment (ExPEC/APEC) were not available for inclusion in this study. Lysis outcomes are reported qualitatively (+/−) under the assay conditions; EOP was not quantified and will be addressed in future work to support cocktail design and application-level translation.

## Data Availability

The original contributions presented in this study are included in the article/Supplementary Material. Further inquiries can be directed to the corresponding author(s).

## Supplementary Materials

The following supporting information can be downloaded at: https://www.mdpi.com/article/10.3390/ijms27031276/s1.

## Abbreviations

The following abbreviations are used in this manuscript:

AMR	Antimicrobial Resistance
BAVS	Bacterial and Archaeal Viruses Subcommittee
BLASTp	Basic Local Alignment Search Tool of Protein
*E. coli*	*Escherichia coli*
ExPEC	Extraintestinal pathogenic *Escherichia coli*
LB	Luria–Bertani broth
NCBI	National Center for Biotechnology Information
MDR	Multidrug-Resistant
OD	Optical density
ORFs	Open Reading Frames
SEM	Scanning Electron Microscope.
TEM	Transmission Electron Microscopy
