# Supplementary material for: A Lytic Mosigvirus Phage (GADS24) from a Poultry-Farm Environment: Genome-Resolved Characterization and In Vitro Biocontrol-Relevant Phenotyping Against Escherichia coli
Source: Int J Mol Sci. 2026 Jan 27;27(3):1276. doi: 10.3390/ijms27031276 (PMC12898486; doi:10.3390/ijms27031276)
Supplement: Supplementary file 1 [file ijms-27-01276-s001.zip › ijms-4087850-supplementary.pdf]

Supplementary Materials Table S1: Genomic features of the GADS24 bacteriophage genome, including locus tags, strand orientation, genomic coordinates, and functional annotations of predicted open reading frames (ORFs). The table highlights key structural, regulatory, and enzymatic proteins involved in phage assembly, replication, DNA metabolism, and host interaction.

locus_tag	Strand	Start	End	Annotation
**GADS24-02**	-	609	1184	Endoribonuclease
**GADS24-06**	-	2321	2734	Endonuclease V N-glycosylase UV repair enzyme
**GADS24-07**	-	2793	3074	Putative internal head protein
**GADS24-08**	-	3071	3559	Glycoside hydrolase family protein
**GADS24-09**	-	3594	4052	RNA pyrophosphohydrolase
**GADS24_28**	-	12211	12287	tRNA-Arg
**GADS24_29**	-	12291	12365	tRNA-Met
**GADS24_34**	-	13872	14159	Internal virion protein
**GADS24_35**	-	14237	14692	RNA ligase
**GADS24_36**	-	14692	14922	Tail fiber assembly protein
**GADS24_38**	-	15715	16299	phage tail protein
**GADS24_39**	-	16394	17215	DNA end protector
**GADS24_40**	-	17212	17862	homing endonuclease
**GADS24_41**	+	17864	18313	head closure
**GADS24_42**	+	18375	18950	baseplate wedge subunit
**GADS24_43**	+	18950	20683	baseplate hub structural protein/lysozyme R
**GADS24_45**	+	21205	21498	PAAR domain-containing protein
**GADS24_46**	+	21507	23480	baseplate wedge subunit
**GADS24_47**	+	23477	26575	baseplate wedge subunit
**GADS24_48**	+	26568	27572	baseplate wedge subunit
**GADS24_49**	+	27646	28518	baseplate wedge tail fiber protein connector
**GADS24_50**	+	28518	30323	baseplate wedge subunit and tail pin
**GADS24_51**	+	30323	30982	baseplate wedge subunit and tail pin
**GADS24_52**	+	30979	32562	tail fiber protein
**GADS24_53**	+	32562	34010	fibritin
**GADS24_54**	+	34043	34969	head-tail adaptor Ad2
**GADS24_55**	+	34971	35735	neck protein
**GADS24_56**	+	35788	36609	tail sheath stabilizer
**GADS24_57**	+	36619	37113	small terminase
**GADS24_58**	+	37097	38932	terminase large subunit
**GADS24_59**	+	38963	40945	tail sheath
**GADS24_60**	+	41059	41550	phage tail protein
**GADS24_61**	+	41635	43206	portal protein
**GADS24_62**	+	43206	43442	prohead core protein
**GADS24_63**	+	43442	43867	prohead core protein
**GADS24_64**	+	43867	44508	head maturation protease
**GADS24_65**	+	44542	45354	head scaffolding protein
**GADS24_66**	+	45372	46940	major head protein
**GADS24_68**	+	47364	48647	capsid vertex protein

**GADS24_69**	-	48682	49680	RNA ligase 2
**GADS24_73**	_	51481	52149	prohead protease inhibitor
**GADS24_74**	+	52200	53714	UvsW helicase
**GADS24_75**	+	53740	53973	DNA helicase
**GADS24_78**	-	54455	54949	UvsY-like recombination mediator
**GADS24_79**	-	54949	55347	putative baseplate wedge subunit
**GADS24_80**	-	55347	55973	gp26 family baseplate hub assembly chaperone
**GADS24_81**	+	56021	56773	baseplate hub assembly protein
**GADS24_82**	+	56770	57942	baseplate hub subunit
**GADS24_83**	+	57890	58423	baseplate hub distal subunit
**GADS24_84**	+	58420	60192	baseplate hub subunit and tail length
**GADS24_85**	+	60201	61310	baseplate tail tube cap
**GADS24_86**	+	61310	62272	baseplate subunit
**GADS24_88**	-	62657	64744	Alt-like RNA polymerase ADP-ribosyltransferase
**GADS24_90**	-	64989	66482	DNA ligase
**GADS24_97**	-	69131	69496	putative base plate hub subunit
**GADS24_100**	-	70614	70862	lysis inhibition; accessory protein
**GADS24_101**	-	70987	71319	head assembly chaperone protein
**GADS24_102**	-	71377	71673	tail fibers protein
**GADS24_103**	-	71675	72256	tRNA-specific adenosine deaminase
**GADS24_104**	-	72256	73248	Phospho-2-dehydro-3-deoxyheptonate aldolase,Tyr-sensitive
**GADS24_107**	-	73951	74175	thymidine kinase
**GADS24_109**	-	74347	75246	polynucleotide kinase
**GADS24_113**	-	76006	76296	Rz-like spanin
**GADS24_114**	-	76293	76649	Rz-like spanin
**GADS24_115**	-	76637	77200	protein Alc
**GADS24_116**	-	77200	78324	RNA ligase
**GADS24_117**	-	78381	78791	endonuclease
**GADS24_118**	-	78818	79996	Ribonucleoside-diphosphate reductase 1 subunit beta
**GADS24_119**	-	80052	82307	Ribonucleoside-diphosphate reductase 1 subunit alpha
**GADS24_121**	-	82651	83511	Thymidylate synthase ThyA
**GADS24_122**	-	83508	84095	dihydrofolate reductase
**GADS24_126**	-	85151	86053	single-stranded DNA-binding protein
**GADS24_127**	-	86171	86824	DNA helicase loader
**GADS24_128**	-	86821	87159	late promoter transcription accessory protein
**GADS24_129**	-	87137	87406	transcriptional regulator
**GADS24_130**	-	87414	88331	RnaseH
**GADS24_131**	+	88436	92311	long tail fiber
**GADS24_132**	+	92320	93447	long-tail fiber protein
**GADS24_134**	+	94181	97237	tail fiber protein
**GADS24_135**	+	97268	97819	tail fiber assembly
**GADS24_136**	+	97829	98488	holin
**GADS24_140**	-	99342	99557	anti-restriction nuclease
**GADS24_144**	-	100623	101255	MotA-like activator of middle period transcription

**GADS24_145**	-	101443	102768	DNA topoisomerase 4 subunit A
**GADS24_147**	-	102913	103059	Ac acridine resistance protein
**GADS24_148**	-	103112	103558	Ndd-like nucleoid disruption protein
**GADS24_151**	-	104418	104894	endonuclease
**GADS24_153**	-	105239	106174	membrane integrity protector
**GADS24_154**	-	106184	108397	rIIA protector from prophage-induced early lysis
**GADS24_157**	-	109846	111663	DNA topoisomerase 4 subunit B
**GADS24_159**	-	112157	112336	FmdB-like transcriptional regulator
**GADS24_161**	-	112749	113021	cef modifier of supressor tRNAs
**GADS24_163**	-	113618	114037	MotB-like transcriptional regulator
**GADS24_166**	-	115168	115845	exonuclease
**GADS24_167**	-	115855	117168	ATP-dependent RecD-like DNA helicase
**GADS24_170**	-	118378	118986	RNA polymerase ADP-ribosylase
**GADS24_171**	-	119044	119625	RNA polymerase ADP-ribosylase
**GADS24_173**	-	119842	120006	molybdenum ABC transporter, periplasmic molybdenum-binding protein
**GADS24_174**	-	120023	120487	Mrh transcription modulator under heat shock
**GADS24_178**	-	121208	121444	capsid and scaffold protein
**GADS24_179**	-	121490	122011	dUTP diphosphatase
**GADS24_181**	-	122294	123316	DNA primase
**GADS24_185**	-	124694	124987	spackle periplasmic protein
**GADS24_186**	-	125051	125299	immunity to superinfection membrane protein
**GADS24_187**	-	125361	125729	immunity protein
**GADS24_189**	-	126004	126216	Dmd discriminator of mRNA degradation
**GADS24_192**	-	127028	128470	replicative DNA helicase
**GADS24_193**	-	128480	128821	head vertex assembly chaperone
**GADS24_194**	-	128814	129986	Protein RecA
**GADS24_195**	-	130086	130634	thymidylate kinase
**GADS24_197**	-	131535	132251	Thymidylate synthase
**GADS24_199**	-	132529	133689	peptidase
**GADS24_200**	-	133749	134135	phosphoheptose isomerase
**GADS24_201**	-	134135	135820	NTP-transferase domain-containing protein
**GADS24_203**	-	136002	136637	Arabinose 5-phosphate isomerase KpsF
**GADS24_204**	-	136678	139389	DNA polymerase
**GADS24_205**	-	139471	139839	translation repressor
**GADS24_206**	-	139842	140405	clamp loader subunit, DNA polymerase accessory protein
**GADS24_207**	-	140407	141369	clamp loader of DNA polymerase
**GADS24_208**	-	141445	142131	putative sliding clamp
**GADS24_209**	-	142174	142590	RNA polymerase binding
**GADS24_210**	-	142603	142791	protein GP45.2
**GADS24_211**	-	142846	144534	endonuclease
**GADS24_214**	-	145011	146030	metallophosphoesterase
**GADS24_219**	-	147018	147575	RNA polymerase sigma factor
**GADS24_229**	-	150073	150381	anaerobic glutaredoxin
**GADS24_231**	-	150711	151181	Anaerobic ribonucleoside-triphosphate reductase-activating protein

**GADS24_232**	-	151178	152995	Anaerobic ribonucleoside-triphosphate reductase
**GADS24_233**	-	152992	153465	endonuclease VII
**GADS24_235**	-	153694	154245	protease inhibitor
**GADS24_238**	-	154761	155024	phage-associated thioredoxin
**GADS24_239**	-	155017	155430	thioredoxin
**GADS24_245**	-	159383	159670	thioredoxin
**GADS24_246**	-	159809	160810	ATP-binding protein
**GADS24_249**	-	161499	162485	nucleotidyltransferase
**GADS24_252**	-	163500	163679	helicase loader
**GADS24_256**	-	164339	164641	lysis inhibition
**GADS24_258**	-	164894	165475	Thymidine kinase
**GADS24_262**	-	166076	166543	phosphatase
**GADS24_263**	-	166536	166883	valyl-tRNA synthetase modifier
**GADS24_265**	-	167431	167889	endoribonuclease
